# Intervenção Coronariana Percutânea em Lesões não Protegidas de Tronco

**DOI:** 10.36660/abc.20190653

**Published:** 2021-06-08

**Authors:** Douglas dos Santos Grion, Debora Carvalho Grion, Igor Veiga Silverio, Leonardo Shingu de Oliveira, Isabela Faria Larini, Anna Victória Martins, Juliana Moreira, Marianne Machado, Lissa Shizue Tateiwa Niekawa, Adriana dos Santos Grion, Cintia Magalhães Carvalho Grion

**Affiliations:** 1 Universidade Estadual de Londrina LondrinaPR Brazil Universidade Estadual de Londrina , Londrina , PR - Brazil; 2 Universidade Federal Fluminense NiteróiRJ Brazil Universidade Federal Fluminense , Niterói , RJ - Brazil; 3 Hospital Evangélico de Londrina LondrinaPR Brazil Hospital Evangélico de Londrina , Londrina , PR - Brazil

**Keywords:** Intervenção Coronária Percutânea/métodos, Doença da Artéria Coronariana, Reperfusão Miocárdica, Angioplastia Coronária com Balão, Stents Farmacológicos/tendências, Ultrassonografia de Intervenção/métodos

## Abstract

**Fundamento:**

O advento dos *stents* farmacológicos permitiu que a intervenção coronariana percutânea apresentasse resultados seguros nas lesões de tronco da artéria coronária esquerda.

**Objetivos:**

Analisar os resultados do tratamento percutâneo da lesão não protegida de tronco da artéria coronária com a utilização de ultrassom intravascular.

**Métodos:**

Estudo de série de casos consecutivos realizado no período de janeiro de 2010 a dezembro de 2018. Coletaram-se dados clínicos dos pacientes, assim como escores prognósticos e dados da lesão coronariana. Considerou-se de sucesso a lesão residual menor que 50% à angiografia e a área mínima da luz maior que 6 mm ^2^ ao ultrassom intravascular. O nível de significância adotado foi de 5%.

**Resultados:**

Analisaram-se 107 casos. A lesão multiarterial foi predominante, sendo com maior frequência (39,25%) encontradas lesões em três vasos além do tronco coronariano. O escore SYNTAX apresentou média de 46,80 (DP: 22,95), e 70 (65,42%) pacientes tiveram escore SYNTAX acima de 32 pontos. Considerou-se sucesso angiográfico da intervenção percutânea em 106 (99,06%) pacientes. A taxa geral de evento maior cardíaco e cerebrovascular no desfecho hospitalar foi 6,54%, sendo semelhante nos pacientes com escore SYNTAX ≤ 32 (8,10%) e ≥ 33 (5,71%; p = 0,68) .

**Conclusões:**

A intervenção percutânea em casos de lesão não protegida de tronco coronariano foi realizada com segurança e apresentou ótimos resultados. Atingiu-se alto sucesso angiográfico de tratamento guiado pelo ultrassom intravascular. A taxa de eventos cardíacos e cerebrovasculares maiores foi semelhante entre os pacientes de menor e de maior risco.

## Introdução

O tratamento das lesões de tronco de artéria coronária esquerda que não estão protegidas por circulação colateral ou *bypass* coronariano permanece um grande desafio para a cardiologia intervencionista atual. O tronco da artéria coronária esquerda irriga grande parte do ventrículo esquerdo em indivíduos com dominância anatômica direita e praticamente a totalidade desse ventrículo na dominância esquerda. Sendo assim, qualquer evento adverso nessa área resulta em alto risco de morbimortalidade. ^1^ Dessa forma, o tratamento clínico pode não ser a melhor opção, e a cirurgia de revascularização miocárdica ainda é a forma mais indicada de tratamento desses pacientes. ^2 , 3^ Em contrapartida, o avanço tecnológico e o advento dos *stents* farmacológicos permitiu que, em casos selecionados, a intervenção coronariana percutânea pudesse apresentar resultados seguros nas lesões de tronco da artéria coronária esquerda. ^4 - 6^

Essa forma de apresentação e de tratamento representa 1% das intervenções coronarianas percutâneas nas síndromes coronarianas agudas, sendo metade em casos de infarto agudo do miocárdio e 70% com envolvimento distal do tronco da artéria coronária esquerda. ^7 , 8^ Todas as formas possíveis de otimização da busca de melhor resultado na intervenção coronariana percutânea devem estar disponíveis. O uso de ultrassom intravascular é recomendado pois auxilia no implante ótimo do *stent* e pode ter impacto na redução de mortalidade. ^9 , 10^

Estudo recente demonstrou não inferioridade ao comparar o tratamento cirúrgico com a angioplastia coronariana com implante de *stent* farmacológico em lesões de artéria coronariana esquerda. A intervenção coronariana percutânea foi relatada como uma alternativa viável e segura à cirurgia de revascularização do miocárdio e pode ser utilizada na prática clínica diária em pacientes selecionados. ^11^ Resultados a longo prazo confirmam que, em pacientes com lesão de tronco de coronária esquerda de baixa a moderada complexidade, a angioplastia tem segurança e eficácia comparável à cirurgia a longo prazo e, consequentemente, constitui uma alternativa válida nesse grupo de pacientes. ^12^

A intervenção coronária percutânea vem sendo cada vez mais utilizada para a revascularização de pacientes com lesões não protegidas de tronco da artéria coronária, e o uso do ultrassom intravascular é descrito com frequência cada vez maior, porém ainda sendo considerado uma recomendação e realizado em uma parcela dos pacientes tratados. ^13^ O objetivo do presente estudo foi analisar os resultados do tratamento percutâneo da lesão não protegida de tronco da artéria coronária com a utilização de ultrassom intravascular.

## Métodos

Estudo de série de casos realizado no período de janeiro de 2010 a dezembro de 2018. Esta pesquisa foi aprovada pelo Comitê de Ética em Pesquisa da Associação Evangélica Beneficente de Londrina, conforme o Parecer nº 2.149.472 de 30 de junho de 2017, CAAE Nº 68385917.0.0000.5696.

Realizou-se o estudo em um laboratório de hemodinâmica de um hospital privado de caráter filantrópico. É um hospital geral de alta complexidade, com 269 leitos, referência no atendimento de urgência e emergência. O laboratório de hemodinâmica dispõe de atendimento contínuo aos pacientes em regime de plantão com equipe de enfermagem e funcionários dimensionada segundo regulamentação nacional vigente. Utilizaram-se *stents* farmacológicos embebidos em sirolimus, everolimus ou biolimus e equipamento de cardiologia intervencionista da marca GE®, e estavam disponíveis exames de ultrassom intravascular das marcas Philips Volcano® e Boston®. Todos os procedimentos do estudo foram guiados por ultrassom intravascular e efetuados pelo primeiro autor deste artigo, por se tratar de hemodinamicista experiente e capacitado para o tratamento dessas lesões coronarianas.

Executou-se amostragem de conveniência dos pacientes adultos submetidos a angioplastia coronariana percutânea por lesão não protegida de tronco coronariano em caráter eletivo de forma consecutiva no período do estudo.

As lesões coronarianas consideradas para indicação da angioplastia coronariana percutânea foram os diagnósticos de angina estável, angina instável, isquemia silenciosa ou infarto agudo do miocárdio sem elevação do segmento ST. Todos os pacientes deveriam ter o diagnóstico recente de estenose não protegida de mais de 50% do diâmetro da artéria coronariana esquerda principal, estimada visualmente, e serem considerados candidatos a procedimento de revascularização do miocárdio. Sucesso da intervenção percutânea foi considerado lesão residual menor que 50% à angiografia e área mínima da luz maior que 6 mm ^2^ ao ultrassom intravascular.

Os dados gerais coletados foram: idade, sexo, datas de internação e de desfecho no hospital, datas de admissão e desfecho na unidade de terapia intensiva (UTI), diagnóstico de admissão hospitalar, presença de doenças crônicas, escore prognóstico Simplified Acute Physiology Score 3 (SAPS 3) ^14^ na admissão da UTI e escore SYNTAX derivado do estudo “SYNergy between percutaneous coronary intervention with TAXus and cardiac surgery”. ^15^ Os dados coletados dos procedimentos de angiográficos foram: número de lesões arteriais detectadas, número de vasos tratados e número de *stents* implantados. Anotaram-se todas as complicações ocorridas no período de acompanhamento intra-hospitalar.

Os eventos cardíacos e cerebrovasculares maiores considerados foram: infarto do miocárdio, acidente vascular encefálico e morte. Acidente vascular encefálico foi definido como déficit neurológico agudo com duração maior que 24 horas. Infarto do miocárdio tipo I, não relacionado ao procedimento, foi definido como elevação de troponina acima do percentil 99 associada a pelo um dos seguintes: sintomas de isquemia aguda do miocárdio, novas alterações isquêmicas no eletrocardiograma, desenvolvimento de ondas Q patológicas ou evidência de nova perda de miocárdio viável ou nova anormalidade regional de movimento de parede em exame de imagem consistente com etiologia isquêmica.

Definiu-se infarto do miocárdio relacionado ao procedimento como elevação dos níveis de troponina maior que cinco vezes acima do percentil 99 até 48 horas após a intervenção percutânea em pacientes com valores basais normais. Em pacientes com valores elevados de troponina antes do procedimento, deve haver elevação acima de 20% do valor basal e o valor absoluto pós-procedimento deve ser pelo menos cinco vezes acima do percentil 99. Além disso, um dos seguintes elementos deve estar presente: novas alterações isquêmicas no eletrocardiograma, desenvolvimento de ondas Q patológicas, evidência de nova perda de miocárdio viável ou nova anormalidade regional de movimento de parede em exame de imagem consistente com etiologia isquêmica ou achados angiográficos consistentes com complicação que limita fluxo coronariano (dissecção coronariana, oclusão de artéria epicárdica ou ramo lateral, limitação de fluxo de colateral ou embolização distal). ^16^

Dividiram-se os pacientes em dois grupos de acordo com o escore SYNTAX para comparação das características clínicas e dos desfechos principais do estudo. O grupo com escore SYNTAX ≤ 32 foi considerado de risco baixo ou intermediário e o grupo com escore ≥ 33 de risco alto para ocorrência de eventos cardíacos e cerebrovasculares maiores.

As fontes utilizadas para a coleta de dados foram o prontuário do paciente e o banco de dados eletrônico do hospital. Coletaram-se os dados por todo o período de permanência hospitalar. Como desfecho principal, consideraram-se os eventos cardíacos e cerebrovasculares maiores até a saída hospitalar.

### Análise estatística

Os dados foram analisados no programa MedCalc Statistical Software versão 15.2.2 (MedCalc Software, Ostend, Belgium). O nível de significância adotado foi de 5% e o intervalo de confiança de 95%.

Na estatística descritiva, as variáveis quantitativas contínuas foram descritas após ser avaliada a aderência à distribuição normal pelo teste Kolmogorov-Smirnov. Para a variável com aproximação à distribuição normal, calcularam-se a média e o desvio padrão (DP) e, caso contrário, a mediana e os intervalos interquartílicos (ITQ) (percentil 25 e percentil 75). Descreveram-se as variáveis categóricas em frequência absoluta e relativa (%).

Na estatística analítica, compararam-se as variáveis categóricas pelo teste exato de Fisher. Para a comparação de dois grupos de variáveis contínuas com amostras independentes, utilizou-se o teste de t de Student para as variáveis com distribuição normal. Para casos cuja distribuição não foi normal, aplicou-se o teste de Mann-Whitney. A mortalidade hospitalar foi descrita como frequência.

## Resultados

Realizou-se a angioplastia de coronariana percutânea por lesão de tronco de artéria coronariana em 107 pacientes no período do estudo, e não houve exclusão de pacientes ( Tabela 1 ). A maior parte dos pacientes apresentava idade maior que 60 anos (75,00%) na entrada do estudo, sendo o sexo masculino o mais frequente (72,89%). O exame de ecocardiografia foi realizado em 57 pacientes, e a fração de ejeção média foi de 53,74% (DP: 10,90).

**Tabela 1:** – Características clínicas dos pacientes

Variável	N	%
Idade, anos (média-DP)	69,05	10,61
Sexo masculino	78,00	72,89
Fração de ejeção (média-DP)	53,74	10,90
Diabetes *mellitus*	61,00	57,01
Hipertensão arterial	90,00	84,11
Hipercolesterolemia	83,00	77,57
Angioplastia prévia	41,00	38,32
IAM prévio	5,00	4,67
Hipotireoidismo	8,00	7,47
Câncer	6,00	5,60
Doença renal crônica dialítica	2,00	1,87
Outras doenças crônicas	3,00	2,80
SAPS 3 (média-DP)	34,78	7,30
Lesão de TCE (%) (média-DP)	65,07	11,76
Lesão distal TCE	53,00	49,53
**Número de vasos doentes**		
TCE	1,00	0,93
TCE + 1 vaso	28,00	26,17
TCE + 2 vasos	36,00	33,64
TCE + 3 ou mais vasos	42,00	39,25
Escore SYNTAX (média-DP)	46,80	22,95
Número de *stents* (média-DP)	3,90	2,33

*DP: desvio padrão; IAM: infarto agudo do miocárdio; SAPS: simplified acute physiology score; TCE: tronco de coronária esquerda.*

A lesão única acometendo somente o tronco de coronária esquerda ocorreu em um paciente. A lesão multiarterial foi predominante na amostra, e com maior frequência (39,25%) foram encontradas lesões em três vasos além do tronco coronariano. As artérias mais frequentemente envolvidas além do tronco coronariano foram 91 (85,04%) casos de artéria descendente anterior, 83 (77,57%) de artéria circunflexa, 50 (46,72%) de artéria coronária direita, 28 (26,26%) de artéria marginal, 24 (22,42%) de artéria diagonal, 16 (14,95%) de artéria descendente posterior e 9 (8,41%) de artéria ventricular posterior. O escore SYNTAX apresentou média de 46,80 (DP: 22,95), e 70 (65,42%) pacientes apresentaram escore SYNTAX acima de 33 pontos ( Tabela 2 ).

**Tabela 2:** – Comparação dos grupos de pacientes de acordo com o escore SYNTAX

Variável	SYNTAX ≤ 32 N = 37	SYNTAX ≥ 33 N = 70	Valor de p
Idade, anos (média-DP)	70,24 (9,79)	68,42 (11,04)	0,40
Sexo masculino N (%)	24,00 (64,86)	54,00 (77,14)	0,25
Fração de ejeção (média-DP)	51,23 (9,21)	55,33 (11,71)	0,18
Diabetes *mellitus* N (%)	20,00 (54,05)	41,00 (58,57)	0,68
Hipertensão arterial N (%)	33,00 (89,19)	57,00 (81,43)	0,40
Hipercolesterolemia N (%)	29,00 (78,38)	54,00 (77,14)	0,54
Angioplastia prévia N (%)	33,00 (89,19)	22,00 (31,43)	0,05
IAM prévio N (%)	2,00 (5,41)	3,00 (4,29)	0,56
SAPS 3 (média-DP)	35,05 (7,34)	34,64 (7,33)	0,78
Lesão de TCE (%) (média-DP)	65,73 (8,20)	64,69 (13,54)	0,74
Lesão distal TCE	18,00 (48,64)	35,00 (50,00)	0,50
Tempo de UTI, dias (mediana-ITQ)	2,00 (1,00 - 4,50)	2,00 (1,50 – 5,00)	0,33
Tempo de hospital, dias (mediana-ITQ)	4,00 (2,50 – 6,50)	3,50 (2,50 – 7,00)	0,87
Evento maior cardíaco e cerebrovascular N (%)	3,00 (8,10)	4,00 (5,71)	0,68
Mortalidade hospitalar N (%)	0 (0,00)	2,00 (2,82)	0,54

*DP: desvio padrão; IAM: infarto agudo do miocárdio; SAPS: simplified acute physiology score; TCE: tronco de coronária esquerda; UTI: unidade de terapia intensiva.*

Considerou-se sucesso angiográfico da intervenção percutânea pelo ultrassom intravascular em 106 (99,06%) pacientes. Em cada procedimento, foram tratadas em média 4,4 (DP: 2,4) lesões e implantados em média 3,9 (DP: 2,3) *stents* . Utilizou-se o ultrassom intravascular em todos os pacientes. A média do lúmen do tronco coronariano medido pelo ultrassom intravascular foi de 4,52 mm ^2^ (DP: 1,05) antes do procedimento de angioplastia, e essa média aumentou para 15,39 mm ^2^ (DP: 3,15) após intervenção percutânea. Em 51 (47,66%) casos, optou-se por realizar procedimentos de forma estadiada. Nesses casos, realizaram-se entre dois e quatro procedimentos para completar o tratamento de todas as lesões coronarianas.

Complicações durante o procedimento ocorreram em 13 pacientes (14,95%), dos quais, 9 apresentaram hematoma em local de punção, sem necessidade de transfusão sanguínea ou intervenção cirúrgica. Dois pacientes tiveram pneumonia hospitalar, um paciente apresentou edema agudo de pulmão e um paciente teve perfuração de artéria coronariana. Neste último caso, realizou-se pericardiocentese de alívio, e o paciente foi encaminhado para drenagem cirúrgica por janela pericárdica. Houve cinco (4,67%) casos de infarto do miocárdio pós-procedimento, sendo todos os casos de infarto relacionados ao procedimento percutâneo, e duas mortes, e não houve acidente vascular encefálico após o procedimento percutâneo durante o período intra-hospitalar de acompanhamento. A frequência de evento maior cardíaco e cerebrovascular no desfecho hospitalar foi 6,54%. Os pacientes permaneceram por tempo mediano de dois dias (ITQ: 1,0 – 5,5 dias) na UTI e quatro dias (ITQ: 2,5 – 7,0 dias) no hospital ( Tabela 3 ).

**Tabela 3:** – Tempo de permanência e desfechos dos pacientes

Variável	N	%
Tempo de UTI, dias (mediana-ITQ)	2	1,00 – 5,50
Tempo de hospital, dias (mediana-ITQ)	4	2,50 – 7,00
Evento maior cardíaco e cerebrovascular	7	6,54
Mortalidade hospitalar	2	1,87

*UTI: unidade de terapia intensiva; ITQ: intervalo interquartílico.*

Ao comparar os pacientes de acordo com o escore SYNTAX, não se observou diferença nas características clínicas ou desfechos relevantes entre o grupo de pacientes com escore elevado e aqueles com escore baixo ou intermediário. As duas mortes relatadas na amostra ocorreram nos pacientes do grupo de escore SYNTAX elevado ( Tabela 2 ). Em um dos casos, a morte foi atribuída a tromboembolismo pulmonar maciço imediatamente após o procedimento de angioplastia percutânea, e o segundo caso foi considerado oclusão coronariana aguda durante o procedimento.

## Discussão

No presente estudo, é relatada experiência com a realização de intervenção percutânea para tratamento de lesão não protegida de tronco coronariano guiada por ultrassom intravascular. Nesse relato de grande número de casos, o procedimento de angioplastia como escolha para tratamento dessas lesões coronarianas complexas mostrou-se seguro com elevado sucesso angiográfico, inclusive para o grupo de pacientes considerado de alto risco.

A otimização da intervenção percutânea com o uso de ultrassom intravascular representa um avanço tecnológico que mudou a prática da cardiologia intervencionista. Além disso, o uso de estratificação de risco pelo escore SYNTAX residual pode ser útil para identificar pacientes que se beneficiam da opção pela intervenção percutânea. ^17^ É objetivo do hemodinamicista atingir expansão ótima do *stent* para minimizar o risco de trombose do *stent* e reestenose. O uso do ultrassom intravascular é componente importante para o sucesso do procedimento. ^18^ Na experiência relatada no presente estudo, utilizou-se o exame de ultrassom intravascular em todos os pacientes para melhor estudo das lesões e avaliação do sucesso angiográfico após o procedimento.

Até pouco tempo, os principais estudos avaliando o uso da intervenção percutânea em lesões não protegidas eram o SYNTAX ^19^ e o PRECOMBAT. ^2^ O desfecho combinado de eventos maiores cardíacos e cerebrovasculares foi semelhante no estudo SYNTAX comparando a intervenção percutânea (36,9%) e a cirurgia de revascularização (31,0%, p = 0,12), assim como a mortalidade de todas as causas. A necessidade de revascularização foi mais frequente nos pacientes alocados para intervenção percutânea, e o acidente vascular encefálico foi mais frequente nos pacientes alocados para cirurgia de revascularização. O estudo PRECOMBAT ^2^ confirmou esses resultados e descreveu maior frequência de isquemia relacionada ao vaso revascularizado no grupo intervenção percutânea. Ambos estudos relatam maior benefício da intervenção percutânea para pacientes com escore SYNTAX ≤ 32.

Mais recentemente outros dois grandes estudos trouxeram novas evidências sobre o assunto. ^20 , 21^ Ambos foram estudos de não inferioridade comparando intervenção percutânea e cirurgia de revascularização para tratar lesão não protegida de tronco coronariano. O estudo EXCEL, que incluiu 1.905 pacientes com lesão de tronco coronariano e risco baixo ou intermediário pelo escore SYNTAX, mostrou não inferioridade da intervenção percutânea comparada à cirurgia de revascularização em todos os desfechos em um período de seguimento de três anos. ^20^ Esse estudo demonstrou que a trombose do *stent* foi menos frequente do que a oclusão do enxerto coronariano. No seguimento de cinco anos do estudo EXCEL, a frequência de eventos maiores permaneceu semelhante entre os grupos. ^22^ Em contrapartida, o estudo NOBLE, que analisou 1.201 pacientes, sugere superioridade da cirurgia aos cinco anos pela necessidade mais frequente de revascularização no grupo da intervenção percutânea. ^21^ Em ambos estudos, a mortalidade aos três ou cinco anos não diferiu entre os dois procedimentos. Os aparentes resultados contraditórios desses dois estudos provavelmente se devem a diferenças dos desfechos primários e de definição de infarto do miocárdio não relacionado ao procedimento entre os estudos. O estudo EXCEL selecionou como desfecho combinado a taxa de mortalidade de todas as causas, o acidente vascular encefálico e o infarto agudo do miocárdio, enquanto o estudo NOBLE ampliou esse desfecho combinado adicionando a necessidade de nova revascularização. No presente estudo, o desfecho combinado assemelha-se ao estudo EXCEL e a baixa taxa de sua ocorrência é concordante com os resultados dos grandes estudos relatados.

Recente metanálise ^23^ incluindo esses grandes estudos sugere que pacientes com lesão não protegida de tronco coronariano submetidos à intervenção percutânea apresentam taxas de ocorrência de acidente vascular encefálico, infarto agudo do miocárdio e morte semelhantes aos pacientes submetidos à cirurgia de revascularização em cinco anos de acompanhamento. Os *stents* farmacológicos mostraram resultados superiores comparados aos *stents* não farmacológicos, e estes últimos não podem mais ser considerados padrão ouro de segurança nas intervenções percutâneas. ^24^ O uso de *stents* farmacológicos de nova geração está associado à menor frequência de complicações pós-procedimento, incluindo a trombose do *stent.* ^25^

O primeiro caso sobre tratamento de lesão de tronco não protegida por intervenção percutânea descrito no Brasil foi o tratamento de um paciente com angina estável e sem contraindicação para cirurgia, no qual se optou por intervenção percutânea realizada com *stent* farmacológico de primeira geração e com bons resultados a curto prazo. ^26^ Outros autores latino-americanos descrevem bons resultados em relatos de casos ou estudos com amostras pequenas de pacientes com escore SYNTAX de baixo ou médio risco. ^27 - 29^ Costantini et al. ^30^ descrevem experiência com 142 pacientes, incluindo 63 casos com SYNTAX de alto risco e com uso de ultrassom intravascular em grande parte dos casos. Os autores apresentaram 81,0% de sucesso avaliado por ultrassom e taxa de mortalidade hospitalar de 1,4%, resultados semelhantes aos encontrados na amostra do presente estudo.

No presente estudo, observou-se alto valor do escore SYNTAX médio de 46,80 comparado aos relatos de literatura. O estudo SYNTAX ^31^ descreveu média de 29 e 30 entre os grupos, o estudo EXCEL estudou pacientes de baixo e médio risco e apresentou média do escore de 20, ^20^ e o estudo NOBLE descreveu média do escore de 22 entre os grupos. ^21^ Dessa forma, é possível inferir que nossos casos apresentam alta complexidade anatômica das lesões coronarianas.

Descrevemos a realização de intervenção percutânea mesmo nos pacientes com escore SYNTAX elevado, com resultados semelhantes aos encontrados em pacientes com risco baixo ou intermediário. Intuitivamente, pacientes com escore SYNTAX acima de 32 deveriam se beneficiar da opção por cirurgia de revascularização, porém esse escore não inclui variáveis clínicas que podem ter grande impacto nos desfechos mensurados. Provavelmente o EuroSCORE tenha melhor desempenho como preditor desses eventos. Outra explicação possível para os resultados semelhantes entre os grupos estudados pode ser a realização de revascularização completa das lesões coronarianas nos pacientes estudados, reduzindo as chances de eventos maiores pós-procedimento. De modo semelhante, outros autores em estudo de centro único não encontraram aumento de mortalidade ou reestenose após três anos de acompanhamento da intervenção percutânea, comparando pacientes com escore SYNTAX baixo-moderado e elevado. ^32^ O risco aumentado de revascularização da lesão culpada em intervenções percutâneas encontrado nos estudos SYNTAX e PRECOMBAT não foi reproduzido nos estudos mais recentes EXCEL e NOBLE.

Na prática clínica, os pacientes com lesão não protegida de tronco coronariano geralmente apresentam maior frequência de comorbidades e piores desfechos quando comparados aos pacientes estudados em grandes ensaios clínicos. Os modelos de predição são ferramentas úteis para auxiliar no planejamento terapêutico dessas lesões coronarianas complexas e otimizar o desfecho dos pacientes pela medicina individualizada. A combinação do escore SYNTAX e do EuroSCORE possivelmente melhora a predição de desfecho na indicação de intervenção percutânea para lesões não protegidas de tronco coronariano. ^33^

Estudos desse tipo apresentam informações que ampliam as indicações de intervenções percutâneas para pacientes selecionados. O perfil periprocedimento de curto tempo de hospitalização, baixas taxas de infecção, baixa necessidade de transfusão sanguínea e custo-efetividade faz a intervenção percutânea muito atrativa. As decisões sobre escolha do procedimento de tratamento devem ser feitas por um time de especialistas considerando características individuais de cada paciente, comorbidades, expectativa de vida, extensão da doença, anatomia angiográfica e preferências do paciente.

Podem ser consideradas limitações deste estudo o delineamento retrospectivo da série de casos, o fato de ser um estudo de centro único e os procedimentos terem sido realizados pelo mesmo profissional hemodinamicista. A generalização dos resultados deve ser feita com cautela para centros com características semelhantes, assim como perfil clínico de pacientes . A maior contribuição do estudo está no uso do ultrassom intravascular em todos os procedimentos e na grande quantidade de casos relatados, que se assemelha ao número de casos de alguns dos grandes ensaios clínicos encontrados na literatura.

## Conclusões

A intervenção percutânea em casos de lesão não protegida de tronco coronariano foi realizada com segurança e apresentou excelentes resultados. Alcançou-se alta taxa de sucesso angiográfico de tratamento guiado pelo ultrassom intravascular. A taxa de eventos cardíacos e cerebrovasculares maiores foi baixa, sendo semelhante entre os pacientes de menor e de maior risco.
