# Supplementary material for: Intervenção Coronariana Percutânea em Lesões não Protegidas de Tronco
Source: Arq Bras Cardiol. 2021 Jun 8;116(6):1101–8. [Article in Portuguese] doi: 10.36660/abc.20190653 (PMC8288543; doi:10.36660/abc.20190653)
Supplement: Translation

# Translation: Percutaneous Coronary Intervention in Unprotected Left Main Coronary Artery Lesions

**Authors:** Douglas dos Santos Grion, Debora Carvalho Grion, Igor Veiga Silverio, Leonardo Shingu de Oliveira, Isabela Faria Larini, Anna Victória Martins, Juliana Moreira, Marianne Machado, Lissa Shizue Tateiwa Niekawa, Adriana dos Santos Grion, Cintia Magalhães Carvalho Grion

**Affiliations:** 1 Brazil Universidade Estadual de Londrina, Londrina, PR - Brazil; 2 Brazil Universidade Federal Fluminense, Niterói, RJ - Brazil; 3 Brazil Hospital Evangélico de Londrina, Londrina, PR - Brazil

**Keywords:** Percutaneous Coronary Intervention/methods, Coronary Artery Diseases, Myocardial Reperfusion, Drug–Eluting Stents/trends, Ultrasonography, Interventional/methods

## Abstract

**Background:**

The advent of drug-eluting stents allowed the percutaneous coronary intervention to present safe results in lesions in the left main coronary artery.

**Objectives:**

To analyze the results of the percutaneous treatment of unprotected left main coronary artery lesion with the use of intravascular ultrasound.

**Methods:**

Study of consecutive case series carried out from January 2010 to December 2018. Clinical data were collected from patients as well as prognostic scores and data on coronary lesion. Low-grade residual lesion (less than 50%) on angiography and minimum luminal area greater than 6 mm^2^on intravascular ultrasound were considered successful. The adopted significance level was 5%.

**Results:**

107 cases were analyzed. The multivessel lesion was predominant, with most (39.25%) of the lesions being found in three vessels in addition to the left main coronary artery. The SYNTAX score had a mean of 46.80 (SD: 22.95), and 70 (65.42%) patients had a SYNTAX score above 32 points. Angiographic success of percutaneous intervention was considered in 106 (99.06%) patients. The overall rate of major cardiac and cerebrovascular events in the hospital outcome was 6.54%, being similar in patients with SYNTAX score ≤ 32 (8.10%) and ≥ 33 (5.71%; p = 0.68).

**Conclusions:**

Percutaneous intervention in cases of unprotected left main coronary artery lesion was safely performed and presented excellent results. Considerable angiographic success of treatment guided by intravascular ultrasound was achieved. The rate of major cardiac and cerebrovascular events was similar between patients at low and high risks.

## Introduction

The treatment of unprotected left main coronary artery lesions by collateral circulation or coronary bypass remains a major challenge for interventional cardiology nowadays. The left main coronary artery irrigates most of the left ventricle in individuals with right coronary dominance and practically this entire ventricle in the left coronary dominance. Therefore, any adverse event in this area results in a high risk of morbidity and mortality.^1^ Thus, clinical treatment may not be the best option, and the myocardial revascularization procedure is still the most appropriate treatment for these patients.^2 , 3^ Conversely, technological advances and the advent of drug-eluting stents allowed, in selected cases, for percutaneous coronary intervention to present safe results for left main coronary artery lesions.^4 - 6^

This form of presentation and treatment represents 1% of percutaneous coronary interventions in acute coronary syndromes, being half cases of acute myocardial infarction and 70% of distal impairment of the left main coronary artery.^7 , 8^ All possibilities for optimizing the search for better results in percutaneous coronary intervention should be available. The use of intravascular ultrasound is recommended, as it assists in the optimal stent implantation and can have an impact on reducing mortality.^9 , 10^

A recent study demonstrated non-inferiority when comparing surgical treatment carried out with coronary angioplasty with drug-eluting stent in left main coronary artery lesions. Percutaneous coronary intervention has been reported as a viable and safe alternative to the myocardial revascularization procedure and can be used in daily clinical practice in selected patients.^11^ Long-term results confirm that, in patients with left main coronary artery lesions of low to moderate complexity, angioplasty is as safe and effective as long-term surgery and, consequently, constitutes a valid alternative for this group of patients.^12^

Percutaneous coronary intervention has been increasingly used for revascularization of patients with unprotected left main coronary artery lesions, and the use of intravascular ultrasound has been increasingly and frequently described, though it is still considered a recommendation and performed in some of the patients who undergo treatment.^13^ The present study aimed at analyzing the results of the percutaneous treatment of unprotected left main coronary artery lesion with the use of intravascular ultrasound.

## Methods

Case series study carried out from January 2010 to December 2018. This research was approved by the Research Ethics Committee of the Associação Evangélica Beneficente de Londrina, according to the Opinion No. 2.149.472 of June 30, 2017, CAAE No. 68385917.0.0000.5696.

The study was carried out in a hemodynamics laboratory from a private philanthropic hospital. It is a general hospital of high complexity, with 269 beds, a reference in urgent and emergency care. The hemodynamics laboratory provides continuous care to patients, with a nursing team and staff under an on-duty regime and dimensioned according to current national regulations. Drug-eluting stents embedded in sirolimus, everolimus, or biolimus and GE® interventional cardiology equipment were used, and Philips Volcano® and Boston® intravascular ultrasound exams were available. All study procedures were guided by intravascular ultrasound and performed by the first author of this article, considering that he is an experienced hemodynamicist and trained for the treatment of these coronary lesions.

Convenience sampling was performed on adult patients who underwent percutaneous coronary angioplasty due to unprotected left main coronary artery lesion on a consecutive basis during the study period.

The coronary lesions considered for indication of percutaneous coronary angioplasty were diagnoses of stable angina, unstable angina, silent ischemia, or acute myocardial infarction without ST-segment elevation. All patients should have a recent diagnosis of unprotected stenosis greater than 50% of the diameter of the left main coronary artery, visually estimated, and be considered candidates for the myocardial revascularization procedure. Success of percutaneous intervention was considered a low-grade residual lesion, of less than 50% on angiography, and a minimum luminal area greater than 6 mm^2^on intravascular ultrasound.

The general data collected were: age, sex, dates of hospitalization and outcome in the hospital, dates of admission and outcome in the intensive care unit (ICU), diagnosis for hospital admission, presence of chronic diseases, prognostic score Simplified Acute Physiology Score 3 (SAPS 3)^14^ on admission to the ICU, and SYNTAX score derived from the study “SYNergy between percutaneous coronary intervention with TAXus and cardiac surgery”.^15^ Data collected from the angiographic procedures were: number of detected arterial lesions, number of treated vessels, and number of implanted stents. All complications that occurred during the intrahospital follow-up period were noted.

The major cardiac and cerebrovascular events considered were: myocardial infarction, cerebrovascular accident, and death. Cerebrovascular accident was defined as an acute neurological deficit lasting more than 24 hours. Type I myocardial infarction, unrelated to the procedure, was defined as an increase in troponin exceeding the 99^th^percentile associated with at least one of the following aspects: symptoms of acute myocardial ischemia, new ischemic changes on the electrocardiogram, development of pathological Q waves, or evidence of new loss of viable myocardium or new regional wall motion abnormality on image examination consistent with ischemic etiology.

Procedure-related myocardial infarction was defined as an increase in troponin levels exceeding more than five times the 99^th^percentile up to 48 hours after percutaneous intervention in patients with normal baseline values. In patients with high troponin values before the procedure, there should be an increase exceeding 20% of the baseline value, and the absolute postoperative value should be at least more than five times the 99^th^percentile. In addition, one of the following elements must be present: new ischemic changes on the electrocardiogram, development of pathological Q waves, evidence of new loss of viable myocardium or new regional wall motion abnormality on imaging examination consistent with ischemic etiology or angiographic findings consistent with a complication that limits coronary flow (coronary artery dissection, occlusion of the epicardial coronary artery or lateral branch, limitation of collateral flow, or distal embolization).^16^

Patients were divided into two groups according to the SYNTAX score for the comparison of clinical characteristics and main study outcomes. The group with SYNTAX score ≤ 32 was considered to be at low or intermediate risk; and the group with score ≥ 33, at high risk for the occurrence of major cardiac and cerebrovascular events.

The sources used for data collection were the patient’s medical record and the electronic database of the hospital. Data were collected throughout the hospital length of stay. As the primary outcome, major cardiac and cerebrovascular events until hospital discharge were considered.

### Statistical analysis

Data were analyzed using the MedCalc Statistical Software, version 15.2.2 (MedCalc Software, Ostend, Belgium). The adopted level of significance was 5% and the confidence interval was 95%.

In descriptive statistics, continuous quantitative variables were described after assessing adherence to normal distribution by the Kolmogorov-Smirnov test. For the variable close to the normal distribution, the mean and standard deviation (SD) were calculated; otherwise, the median and interquartile ranges (IQR) (25^th^percentile and 75^th^percentile) were considered. Categorical variables were described in absolute and relative frequencies (%).

In analytical statistics, categorical variables were compared using Fisher’s exact test. To compare two groups of continuous variables with independent samples, Student’s t-test was used for variables with normal distribution. For cases with non-normal distribution, the Mann-Whitney test was considered. Hospital mortality was described as frequency.

## Results

Percutaneous coronary angioplasty was performed due to left main coronary artery lesion in 107 patients during the study period, and no patient was excluded ( Table 1 ). Most patients aged over 60 years (75.00%) at the beginning of the study, with prevalence of men (72.89%). Echocardiogram was performed in 57 patients, and the mean ejection fraction was 53.74% (SD: 10.90).

**Table 1:** – Clinical characteristics of patients

Variable	N	%
Age, years (mean-SD)	69.05	10.61
Men	78.00	72.89
Ejection fraction (mean-SD)	53.74	10.90
Diabetes mellitus	61.00	57.01
Arterial hypertension	90.00	84.11
Hypercholesterolemia	83.00	77.57
Previous angioplasty	41.00	38.32
Previous AMI	5.00	4.67
Hypothyroidism	8.00	7.47
Cancer	6.00	5.60
Chronic kidney disease requiring dialysis	2.00	1.87
Other chronic diseases	3.00	2.80
SAPS 3 (mean-SD)	34.78	7.30
LMCA lesion (%) (mean-SD)	65.07	11.76
Distal LMCA lesion	53.00	49.53
**Number of affected vessels**		
LMCA	1.00	0.93
LMCA + 1 vessel	28.00	26.17
LMCA + 2 vessels	36.00	33.64
LMCA + 3 vessels or over	42.00	39.25
SYNTAX score (mean-SD)	46.80	22.95
Number of stents (mean-SD)	3.90	2.33

*SD: standard deviation; AMI: acute myocardial infarction; SAPS: Simplified Acute Physiology Score; LMCA: left main coronary artery.*

The single lesion only affecting the left main coronary artery was found in one patient. The multivessel lesion was predominant, with most (39.25%) of the lesions being found in three vessels in addition to the left main coronary artery. The most frequently impaired arteries, in addition to the left main coronary artery, were 91 (85.04%) cases of anterior descending artery; 83 (77.57%) of the circumflex artery; 50 (46.72%) of the right coronary artery; 28 (26, 26%) of marginal artery; 24 (22.42%) of diagonal artery; 16 (14.95%) of posterior descending artery; and 9 (8.41%) of posterior interventricular artery. The SYNTAX score had a mean of 46.80 (SD: 22.95), and 70 (65.42%) patients had a SYNTAX score above 33 points ( Table 2 ).

**Table 2:** – Comparison of groups of patients according to the SYNTAX score

Variable	SYNTAX ≤ 32 N = 37	SYNTAX ≥ 33 N = 70	p-value
Age, years (mean-SD)	70.24 (9.79)	68.42 (11.04)	0.40
Men N (%)	24.00 (64.86)	54.00 (77.14)	0.25
Ejection fraction (mean-SD)	51.23 (9.21)	55.33 (11.71)	0.18
Diabetes mellitus N (%)	20.00 (54.05)	41.00 (58.57)	0.68
Arterial hypertension N (%)	33.00 (89.19)	57.00 (81.43)	0.40
Hypercholesterolemia N (%)	29.00 (78.38)	54.00 (77.14)	0.54
Previous angioplasty N (%)	33.00 (89.19)	22.00 (31.43)	0.05
Previous AMI N (%)	2.00 (5.41)	3.00 (4.29)	0.56
SAPS 3 (mean-SD)	35.05 (7.34)	34.64 (7.33)	0.78
LMCA lesion (%) (mean-SD)	65.73 (8.20)	64.69 (13.54)	0.74
Distal LMCA lesion	18.00 (48.64)	35.00 (50.00)	0.50
ICU length of stay, days (median-IQR)	2.00 (1.00 – 4.50)	2.00 (1.50 – 5.00)	0.33
Hospital length of stay, days (median-IQR)	4.00 (2.50 – 6.50)	3.50 (2.50 – 7.00)	0.87
Major cardiac and cerebrovascular event N (%)	3.00 (8.10)	4.00 (5.71)	0.68
Hospital mortality N (%)	0 (0.00)	2.00 (2.82)	0.54

*SD: standard deviation; AMI: acute myocardial infarction; SAPS: Simplified Acute Physiology Score; LMCA: left main coronary artery; ICU: intensive care unit.*

Angiographic success of percutaneous intervention by intravascular ultrasound was considered in 106 (99.06%) patients. In each procedure, a mean of 4.4 (SD: 2.4) lesions were treated, and a mean of 3.9 (SD: 2.3) stents were implanted. Intravascular ultrasound was used in all patients. The mean coronary artery lumen diameter measured by intravascular ultrasound was 4.52 mm^2^(SD: 1.05) before the angioplasty procedure, and this mean increased to 15.39 mm^2^(SD: 3.15) after percutaneous intervention. In 51 (47.66%) cases, it was decided to perform staged procedures. In these cases, between two and four procedures were performed to complete the treatment of all coronary lesions.

Complications during the procedure occurred in 13 patients (14.95%), of whom 9 presented hematoma at the puncture site, without the need for blood transfusion or surgical intervention. Two patients had hospital-acquired pneumonia, one patient had acute pulmonary edema, and one patient had coronary artery perforation. In the latter case, pericardiocentesis was performed, and the patient was referred for surgical drainage via the pericardial window. There were five (4.67%) cases of postoperative myocardial infarction, all cases of infarction being related to the percutaneous procedure, and two deaths; there was no stroke after the percutaneous procedure during the intrahospital follow-up period. The frequency of major cardiac and cerebrovascular events in the hospital outcome was 6.54%. Patients had a median length of stay of two days (IQR: 1.0 – 5.5 days) in the ICU and four days (IQR: 2.5 – 7.0 days) in the hospital ( Table 3 ).

**Table 3:** – Length of hospital stay and patients’ outcomes

Variable	N	%
ICU length of stay, days (median-IQR)	2	1.00 – 5.50
Hospital length of stay, days (median-IQR)	4	2.50 – 7.00
Major cardiac and cerebrovascular event	7	6.54
Hospital mortality	2	1.87

*ICU: intensive care unit; IQR: interquartile range.*

When comparing patients according to the SYNTAX score, there was no difference in clinical characteristics or relevant outcomes between the group of patients with high score and those with low or intermediate score. The two deaths reported in the sample occurred in patients in the high SYNTAX score group ( Table 2 ). In one case, death was attributed to massive pulmonary embolism immediately after the percutaneous angioplasty procedure, and the second case was considered to be due to acute coronary occlusion during the procedure.

## Discussion

In the present study, experience with the performance of percutaneous intervention for the treatment of unprotected left main coronary artery lesion guided by intravascular ultrasound is reported. In this report of a large number of cases, the angioplasty procedure as a choice for the treatment of these complex coronary lesions proved to be safe and with high angiographic success, including for the group of patients considered to be at high risk.

The optimization of percutaneous intervention with the use of intravascular ultrasound represents a technological advance that has changed the practice of interventional cardiology. In addition, the use of risk stratification by the residual SYNTAX score can be useful to identify patients who benefit from the option for percutaneous intervention.^17^ Hemodynamicists aim at achieving optimal expansion of the stent in order to minimize the risk of stent thrombosis and restenosis. The use of intravascular ultrasound is an important component for the success of the procedure.^18^ In the experience reported in the present study, intravascular ultrasound was used in all patients for better studying the lesions and assessing angiographic success after the procedure.

Until recently, the main studies evaluating the use of percutaneous intervention in unprotected lesions were SYNTAX^19^ and PRECOMBAT.^2^The composite outcome of major cardiac and cerebrovascular events was similar in the SYNTAX study comparing percutaneous intervention (36.9%) and myocardial revascularization procedure (31.0%, p = 0.12) as well as mortality from all causes. The need for myocardial revascularization was more frequent in patients allocated for percutaneous intervention, and stroke was more frequent in patients allocated for the revascularization procedure. The PRECOMBAT^2^study confirmed these results and described a higher frequency of ischemia related to the revascularized vessel in the percutaneous intervention group. Both studies report a greater benefit of percutaneous intervention for patients with SYNTAX score ≤ 32.

More recently, two other large studies have brought new evidence on the subject.^20 , 21^ Both were non-inferiority studies comparing percutaneous intervention and myocardial revascularization procedure to treat unprotected left main coronary artery lesion. The EXCEL study, which included 1,905 patients with left main coronary artery lesion and at low or intermediate risk by the SYNTAX score, showed non-inferiority of percutaneous intervention compared with the myocardial revascularization procedure in all outcomes over a three-year follow-up period.^20^ This study demonstrated that stent thrombosis was less frequent than coronary graft occlusion. In the five-year follow-up period of the EXCEL study, the frequency of major events remained similar between groups.^22^ Conversely, the NOBLE study, which analyzed 1,201 patients, suggests superiority of the procedure at five years of follow up due to the more frequent need for myocardial revascularization in the percutaneous intervention group.^21^ In both studies, mortality at three or five years of follow up did not differ between the two procedures. The apparent contradictory results of these two studies are probably due to differences in primary outcomes and definition of myocardial infarction unrelated to the procedure between studies. The EXCEL study selected as composite outcome the mortality rate from all causes, cerebrovascular accident, and acute myocardial infarction, whereas the NOBLE study expanded this outcome by adding the need for a new revascularization. In the present study, the composite outcome is similar to the EXCEL study, and the low rate of its occurrence is consistent with the results of the large studies reported.

A recent meta-analysis23 including these large studies suggests that patients with unprotected left main coronary artery lesion undergoing percutaneous intervention have rates of occurrence of stroke, acute myocardial infarction, and death similar to patients undergoing the myocardial revascularization procedure in five years of follow up. Drug-eluting stents have shown superior results compared with nondrug-eluting stents, and the latter can no longer be considered the gold standard of safety in percutaneous interventions.^24^ The use of new-generation drug-eluting stents is associated with a lower frequency of postoperative complications, including stent thrombosis.^25^

The first case on treatment of unprotected left main coronary artery lesion by percutaneous intervention described in Brazil was the treatment of a patient with stable angina and without contraindication for surgery, in which percutaneous intervention was performed with first-generation drug- eluting stent and presented good results in the short-term.^26^ Other Latin American authors describe good results in case reports or studies with small samples of patients with SYNTAX score graded as low or intermediate risk.^27 - 29^ Costantini et al.^30^ describe an experience with 142 patients, including 63 cases with high-risk SYNTAX score and with the use of intravascular ultrasound in most cases. The authors verified 81.0% of success assessed by ultrasound and a hospital mortality rate of 1.4%, results similar to those found in the sample of the present study.

In the present study, a high value was found for the mean SYNTAX score (46.80) compared with reports in the literature. The SYNTAX^31^ study described a mean of 29 and 30 between groups; the EXCEL study evaluated low- and intermediate-risk patients and had a mean score of 20;^20^ and the NOBLE study described a mean score of 22 between groups.^21^ Thus, it is possible to infer that the present cases have a high anatomical complexity of coronary lesions.

The performance of percutaneous intervention is described even in patients with a high SYNTAX score, with results similar to those found in patients with low or intermediate risk. Intuitively, patients with a SYNTAX score above 32 should benefit from the option for myocardial revascularization procedure, but this score does not include clinical variables that can have a major impact on the measured outcomes. The EuroSCORE is likely to perform better as a predictor of these events. Another possible explanation for the similar results between the studied groups may be the performance of complete revascularization of the coronary lesions in the studied patients, reducing the chances of major postoperative events. Similarly, other authors in a single-center study did not find an increase in mortality or restenosis after three years of follow up of the percutaneous intervention, comparing patients with low-intermediate and high SYNTAX scores.^32^ The increased risk of culprit-lesion revascularization in percutaneous interventions found in the SYNTAX and PRECOMBAT studies has not been reproduced in the more recent EXCEL and NOBLE studies.

In clinical practice, patients with unprotected left main coronary artery lesion generally have more comorbidities and worse outcomes when compared with patients evaluated in large clinical trials. Prediction models are useful tools to assist in the therapeutic planning of these complex coronary lesions and to optimize the outcome of patients through individualized medicine. The combination of the SYNTAX score and the EuroSCORE possibly improves the outcome prediction as for the indication to percutaneous intervention for unprotected left main coronary artery lesions.^33^

Studies of this type present information that broadens the indications to percutaneous interventions for selected patients. The periprocedural profile of short hospital stay, low rates of infection, reduced need for blood transfusion, and cost-effectiveness make percutaneous intervention very attractive. Decisions regarding the choice of treatment procedure must be made by a team of specialists considering each patient’s individual characteristics, comorbidities, life expectancy, disease extension, angiographic anatomy, and preferences.

As limitations of this study, the retrospective design of the case series, the fact that it is a single-center study, and the procedures being performed by the same hemodynamicist could be considered. The generalization of results should be carefully done for centers with similar characteristics as well as the clinical profile of patients. The greatest contribution of the study is the use of intravascular ultrasound in all procedures and the large number of reported cases, which is similar to the number of cases of some of the large clinical trials found in the literature.

## Conclusions

Percutaneous intervention in cases of unprotected left main coronary artery lesions was safely performed and presented excellent results. Considerable angiographic success of treatment guided by intravascular ultrasound was achieved. The rate of major cardiac and cerebrovascular events was low and similar between patients at low and high risks.
